# Magnetic MoS_2_ pizzas and sandwiches with Mn_n_ (n = 1–4) cluster toppings and fillings: A first-principles investigation

**DOI:** 10.1038/srep19504

**Published:** 2016-01-18

**Authors:** Meng Zhang, Zhongjia Huang, Xiao Wang, Hongyu Zhang, Taohai Li, Zhaolong Wu, Youhua Luo, Wei Cao

**Affiliations:** 1 Department of Physics, East China University of Science and Technology, Shanghai 200237, China; 2 School of Mechanical and Automotive Engineering, Anhui Polytechnic University, Wuhu 241000, China; 3 College of Chemistry, Key Lab of Environment Friendly Chemistry and Application in Ministry of Education, Xiangtan University, Xiangtan, 411105, China; 4 Research Centre for Molecular Materials, University of Oulu, P.O. Box 3000, FIN-90014, Finland

## Abstract

The inorganic layered crystal (ILC) MoS_2_ in low dimensions is considered as one of the most promising and efficient semiconductors. To enable the magnetism and keep intrinsic crystal structures, we carried out a first-principles study of the magnetic and semiconductive monolayer MoS_2_ adsorbed with the Mn_n_ (n = 1–4) clusters, and bilayer MoS_2_ intercalated with the same clusters. Geometric optimizations of the Mn_n_@MoS_2_ systems show the complexes prefer to have Mn_n_@MoS_2_(M) pizza and Mn_n_@MoS_2_(B) sandwich forms in the mono- and bi-layered cases, respectively. Introductions of the clusters will enhance complex stabilities, while bonds and charge transfers are found between external Mn clusters and the S atoms in the hosts. The pizzas have medium magnetic moments of 3, 6, 9, 4 μ_B_ and sandwiches of 3, 2, 3, 2 μ_B_ following the manganese numbers. The pizzas and sandwiches are semiconductors, but with narrower bandgaps compared to their corresponding pristine hosts. Direct bandgaps were found in the Mn_n_@MoS_2_(M) (n = 1,4) pizzas, and excitingly in the Mn_1_@MoS_2_(B) sandwich. Combining functional clusters to the layered hosts, the present work shows a novel material manipulation strategy to boost semiconductive ILCs applications in magnetics.

Since a pioneering (re)discovery of the monolayer graphene^1^,^2^, enormous researches have been focused on layered crystals during the past decades^3^. Benefiting from covalence bonds in layers and van der Waals forces among layers, complex structures are easily manipulated in order to reach different application purposes. In contrast to the semimetallic pristine graphene, most of the inorganic layered crystals (ILCs) have non-zero bandgaps. Such semiconducting properties are essential in semiconductor industry. Various species from the nitrides and group VI metal compounds^4^ enrich the library of the ILCs. Among them, the MoS_2_ is a conventional lubricant^5^ and catalyst for hydrogen evolution^6^,^7^. When lowering the MoS_2_ dimensions, a transition of indirect to direct band occurs, leading to the boosting of photoluminescence^8^,^9^. Recent investigations of the materials in low dimensions showed that it is advanced in high efficient transistors^10^, photoelectronic devices^11^, and electrocatalysis^12^. To improve the performances of the MoS_2_-based devices, molecular modulation and engineering are proposed and many of works are in progresses^13^,^14^. Ideally speaking, the ILC materials can be piled up to 3D forms, with each 2D layer consisting of enough functional units such as doped atoms or clusters^4^. So is the few-layer MoS_2_, which has potentials in various applications and was reported as a key material in high mobility and low power transistors^10^,^15^.

Pursuing semiconductors with high performances has always been within the focuses of materials sciences. Due to possible manipulations of electron spins and carrier intensities^16^, dilute magnetic semiconductors (DMSs) have been one of the key targets within such an issue. Similar to the hosting merits from the transition metal (TM) oxides (say the ZnO)^17^, the structural uniqueness of ILCs makes such layered groups as new candidate matrixes in the magnetic semiconductors. The 2D DMS systems have been explored in monolayer MoS_2_ e.g., by substituting Mo ions with 3*d* TMs^18^,^19^ or 4*d* TMs^20^. The doping routes in the ILCs are, nevertheless, not an easy task in experiments^21^. Alternatives were suggested to replace both Mo and neighboring S atoms with the FeX_6_ (X = S, C, N, O, F) clusters^22^, or a wetting deposition of the Co layer onto monolayer MoS_2_^23^. Brewing magnetism into double- or few-layered MoS_2_ are rather scarcely reported, despite of the magnification of efficiencies in the 3D piled-up electronics^4^. In a latest work, the Fe doped double layered MoS_2_ was predicted to enhance the host stabilities as well as to magnetically exchange coupling between the host and dopants^24^. However, doping or growing dynamics of such hetero functional units normally debuts from a fast nucleation of the metallic ions^25^,^26^, resulting in rather small clusters or nanoparticles on the layered crystal surfaces or possibly among the layers^27^,^28^,^29^. Indeed, in addition to clusters’ inimitable properties^30^,^31^, combining clusters with the monolayer graphene was predicted to increase the magnetic moment of the cluster^32^,^33^,^34^. Furthermore, the intercalated water molecules between graphene interlayers were observed experimentally and very unique properties have been revealed recently^35^. Thus, in an analogy to routes of clusters anchoring on the graphene, using the clusters as dopants onto or among the ILCs may offer another effective route to tailor the ILCs properties.

In this article, we reported on a first-principles prediction of magnetic monolayer MoS_2_ ‘pizzas’ with Mn_n_ (n = 1–4) ‘toppings’, and bilayer MoS_2_ ‘sandwiches’ with Mn_n_ (n = 1–4) clusters ‘fillings’. The manganese clusters were selected as the start clusters due to their magnetic robustness^36^, size-dependent magnetism^37^, as well as easier adsorptions on layered structures^38^. Hosts of the clusters were extended from monolayer MoS_2_ ‘crust’ to the bilayer ‘bread slices’. To keep the intrinsic layered structures and prevent introducing defects, the clusters were placed and adsorbed on the top or between the layers. We investigated magnetic properties and electronic structures of the cluster adsorbed mono- and bi-layers. Bonding mechanisms within Mn clusters, and between clusters and ILC hosts were studied. In addition to possible variations of magnetic moments by changing the cluster sizes, we also found that introductions of the clusters into the ILC systems will facilitate system stabilities, and operate band types.

## Results

### Geometric structures of the complexes

**Figure 1:**
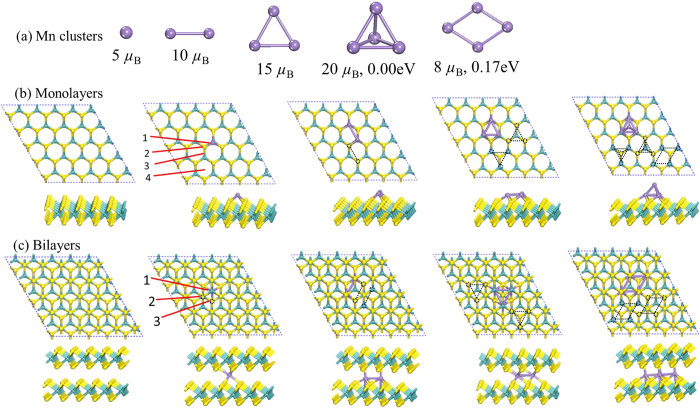
Optimized geometric structures. (**a**) The Mn_n_ (n = 1–4) clusters, (**b**) the Mn_n_ clusters adsorbed on monolayer, and (**c**) bilayer MoS_2_ from the top view (up) and the side view (below). The blue, yellow and purple spheres represent the Mo, S and Mn atoms, respectively. Possible adsorption sites are labelled in numbers in (**b**,**c**). The dash-dot ball and line refer to metastable positional alignments of the Mn_n_ clusters adsorbed on the MoS_2_ host upon geometry optimization.

Investigations of the doped system’s properties debut from the geometric structures. The free Mn_n_ clusters were firstly studied to give basic knowledge of the ‘toppings’ or ‘fillings’ onto or into the crusts or bread slices. More than 60 initial Mn_n_ structures were collected for further DFT optimization. Optimized clusters geometries were depicted in Fig. 1a. Detailed structural parameters for other low-lying isomers are provided in the Supplementary Information (SI). The Mn_n_ clusters evolved from 0D to 2D forms when n was tuned from 1 to 3. However, in the case of n = 4, the 3D tetrahedron cluster was found as the most stable isomer, followed by a rhombus with a relative energy of 0.17 eV higher. Our results of free Mn clusters are in agreement with the previous DFT calculations^39^,^40^,^41^.

From the hosts’ side, the 5 × 5 supercells of single- and two-layer MoS_2_ were firstly relaxed and the optimized results were shown on the left row in Fig. 1b,c. The lattice constants are 3.21 and 3.20 Å for the monolayer and bilayer MoS_2_, respectively, in good agreements with other density functional theory (DFT) calculations^42^. In general, the energy and magnetic properties of the Mn_n_@MoS_2_ systems are sensitive to coordination and contact positions of the Mn_n_ clusters with respect to the ILC hosts. In what follows, we name the systems of Mn_n_@MoS_2_(M) for the Mn_n_ cluster doped Monolayer complexes, and Mn_n_@MoS_2_(B) for the Bilayer ones. After a full optimization of all possible motifs including magnetic order effects, it is found that the Mn_n_@MoS_2_(M) prefer pizza structures. Manganese clusters maintain their initial geometries after being adsorbed on the MoS_2_ monolayer. However, the Mn clusters evolve to a parallel layer motif when intercalated in the bilayers. The Mn_n_@MoS_2_(B) have sandwich structures with the maximal coordination numbers to expand the interactions between the Mn_n_ clusters and two layers of the MoS_2_.

Geometric arrangements of the Mn_n_ clusters and their hosts were studied in details. For the Mn_n_@MoS_2_(M), the most stable adsorption sites of the Mn atoms are right above the Mo atom (numbered 1 in Fig. 1b) from the top view. Other possible adsorption sites on-top S atom, bridge and face as labelled 2, 3, and 4 in Fig. 1b are energetically unfavorable. Our optimizations show that each Mn adatom is bonded to three S atoms at the monolayer MoS_2_ site. For the Mn_4_@MoS_2_(M), the most stable structure is constructed by the tetrahedron Mn_4_ cluster whose triangular face lays on the MoS_2_ host. The rhombic structure with four Mn atoms tiled above the Mo site is less stable with higher energy as shown in Fig. 1b. On the contrary, the structure of the three-dimensional tetrahedron Mn_4_ cluster sandwiched between two layers of the MoS_2_ is unstable according to our calculation. In the figures, the dash-dot balls and lines refer to metastable positions of the Mn_n_ clusters on the monolayer MoS_2_. However, in the case of inserting Mn_n_ to bilayer MoS_2_ systems, the lowest energy structures exhibit odd-even alternations with the number of the Mn atoms. One Mn atom laying under the Mo atom (labelled 1 in Fig. 1c) is the lowest energy structures of odd number n with each adatom Mn bonding six S atoms in the bilayer MoS_2_, while the Mn atoms preferring to be under the S atom (featured 2 in Fig. 1c) and forming the most stable Mn_n_@MoS_2_(B) (n = 2,4) complexes. In this case of evenly numbered n, each Mn atom is bonded to four host S atoms and one Mo atom as Fig. 1c shows.

### Bonding scheme and stability

Table 1 gives bond lengths of the Mn_n_ clusters adsorbed on the monolayer and bilayer MoS_2_ at the most stable adsorption sites. Two kinds of hosting atoms are classified: participators with whom dopants are bonded, and spectators where no additional bonding are formed. The MoS_2_ honey comb structures remain unchanged after absorptions of the Mn_n_ clusters. Very slight lattice distortions are found at the participator area near the Mn_n_ clusters. From Table 1, it can be seen that the distances of the *d*_S–Mo_ between the participator atoms of the MoS_2_ became larger than those of the pristine MoS_2_ in both the ‘pizza’ and ‘sandwich’ cases. These participator S atoms interact with the impurity Mn_n_ clusters, weakening bonding between the S and Mo atoms. On the contrary, the bond length of spectator atoms is the same as the one of the pristine MoS_2_. The Mn-Mn bond lengths in ‘pizzas’ and ‘sandwiches’, except for the Mn_3_@MoS_2_(M) and Mn_4_@MoS_2_(M), are notably larger than those in the free Mn_n_ clusters, also indicating a covalent-bond interaction between the Mn and S atoms. The Mn-Mo bond lengths in Mn_2_@MoS_2_(B) and Mn_4_@MoS_2_(B) are 2.762 and 2.800 Å, respectively. Notably, values of the farthest *d*_Mo-S_ in Mn_n_@MoS_2_(B) oscillate with the Mn numbers. The oscillating trend pervades to electronic and magnetic properties of the Mn_n_@MoS_2_ (B) sandwiches in the follow discussion.

**Figure 2:**
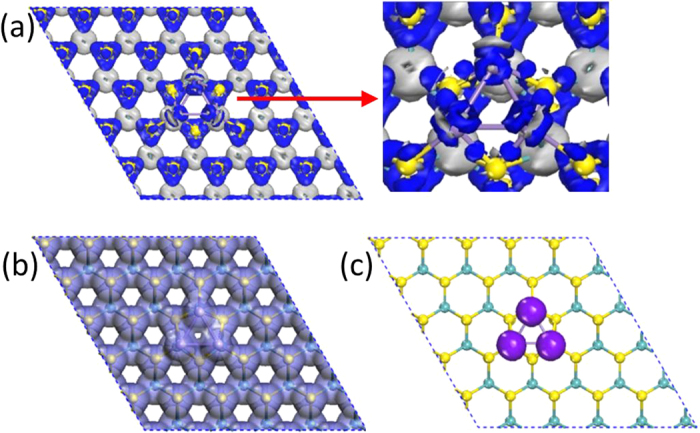
Electron densities of the Mn_3_@MoS_2_(M) complex. (**a**) The deformation electron density (DED). Charge accumulations are obvious in blue regions and depletions in silvery regions. (**b**) Total electron density. (**c**) The net spin electron density. The surface isovalue for electron density is 0.04 e/Å^3^.

The adsorption energies *E*_*ad*_ of the Mn_n_ clusters adsorbed on the ILC hosts were computed as follows.


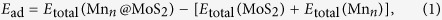


where *E*_total_(Mn_n_@MoS_2_) and *E*_total_(MoS_2_) represent the total energies of the lowest-energy structures of the adsorbates and pristine MoS_2_, respectively, and *E*_total_(Mn_n_) is the energy of the individual Mn_n_ clusters. All adsorption energies are found largely below zero (see Table 1), indicating stability of MoS_2_ after the introductions of the Mn_n_ clusters. Obviously, the absolute value of *E*_*ad*_ increases with the numbers of the Mn atoms due to the increase of the covalent-bond interaction between the Mn and S atoms. To have a better view of the interactions between the Mn_n_ clusters and MoS_2_ layers, the deformation electron density (DED) of the Mn_3_@MoS_2_(M) for the lowest-energy structures was plotted in Fig. 2a as an example. The DED is defined as the total charge density of a system with the density of the isolated atoms subtracted. The blue and silvery area indicate electron accumulation and depletion when atoms forming the Mn_3_@MoS_2_(M). When the Mn_3_ cluster is adsorbed on the MoS_2_ slab, the DED distributes not only surrounding the Mo and S atoms in the host MoS_2_ but also remarkably at the intervals between the Mn and S atoms and the guest Mn clusters (see Fig. 2a). Featuring covalent characters of the S-Mn bonds, the DED identifies strong interactions between the Mn and S in the Mn_n_@MoS_2_(M&B) and high stability of the structure due to such interactions.

### Magnetic properties

The lowest-spin arrangements of individual clusters are all ferromagnetic from our present calculations. Magnetic moments of the Mn_n_ (n = 1–4) clusters are 5, 10, 15, and 20 μ_B_, respectively, in agreement with previous studies^40^,^41^,^42^. It is important to understand the host influence on the magnetic orders of the magnetic guests. For this purpose, we optimized all magnetic spin states of the lowest-energy structures of the ‘pizzas’ and ‘sandwiches’ from Fig. 1b,c. Table 2 gives the relative energies with respect to the most stable spin states of the Mn_n_ clusters adsorbed on the ILC hosts at the lowest energy adsorption sites. Results show that magnetic moments of the impurity Mn_n_ clusters are not quenched by the nonmagnetic host MoS_2_ substrate. The energetic magnetic spin state displays ferrimagnetic properties when ferromagnetic Mn clusters adsorbed on the MoS_2_. The Mn_n_@MoS_2_(M) pizzas prefer to have medium magnetic moments of 3, 6, 9, and 4 μ_B_ in comparison with their corresponding free Mn_n_ clusters (5, 10, 15, 20 μ_B_). The Mn_n_@MoS_2_(B) sandwiches exhibit favorable oscillatory behavior with relatively smaller magnetic moments of 3, 2, 3, and 2 μ_B_. To reveal detailed contributions from each Mn atom in the ‘topping’ or ‘fillings’, we also studied the local spin state on the Mn atom of the Mn_n_@MoS_2_ (M&B) systems. Their magnetic moments are listed in Table S1 (see details in SI). The Mn atoms in Mn_n_@MoS_2_(M) pizzas are in ferromagnetic states except for theses of the Mn_4_@MoS_2_(M). On the Mn_4_@MoS_2_(M) pizza, three tiled Mn atoms have “spin-up” (majority) magnetic moments and one top Mn atom has “spin-down” (minority) magnetic moments. While in the Mn_n_@MoS_2_(B) sandwiches, the Mn atoms display ferrimagnetic order as shown in Table S1. Thus the guest Mn_n_ clusters may serve as an ideal system to tailor magnetic properties when introduced on or between the MoS_2_ ‘crust’ or ‘bread slices’. Continued experimental and theoretical studies of similar TM clusters adsorbed on MoS_2_ systems may lead to discoveries of new families of dilute magnetic semiconductors with tunable magnetic properties.

It should be noted that the magnetic properties of the Mn_7_ cluster absorbed on graphene exhibits a magnetic moment of 6.3 μ_B_ per cell as given by first-principles calculations^32^. This value is 1.3 μ_B_ larger than 5.0 μ_B_ in an isolated Mn_7_ cluster. In the case of Mn doped MoS_2_ studied through a combination of DFT calculations and Monte Carlo simulations, the overall magnetic moment of the supercell is 1 μ_B_ corresponding to the single excess *d* electron provided by the Mn atom^43^. On the other hand, magnetic properties of nonmetal atoms adsorbed MoS_2_ monolayers were also investigated by first-principles calculations. The total magnetic moments of H-,B-, C-, N-, and F-adsorbed MoS_2_ monolayers were found 1, 1, 2, 1, and 1 μ_B_, respectively^44^. The magnetic motifs of all these three cases are different from that of the Mn_n_@MoS_2_ ‘pizzas’ and ‘sandwiches’ studied here. By comparing the magnetic properties between other cases and this work, more insights may be provided into the effect of the impurities types employed on a nonmagnetic layer host.

Mulliken population analysis shows that the total magnetic moment of the clusters is mainly localized in the Mn atoms as tabulated in Table 3. A small amount of magnetic moment is found in host Mo and S atoms. To visualize the spin distribution of the Mn_n_@MoS_2_(M) ‘pizza’, the isosurface spin density of the ‘pizza’ was plotted in Fig. 2. It can be seen from Fig. 2b,c that although the total charge density is extended over the whole Mn_3_@MoS_2_(M), the spin density is almost entirely located on the Mn_3_ cluster site, resulting in a robust magnetic moment of 9 μ_B_ for the Mn_3_@MoS_2_(M).

### Electronic structures

**Figure 3:**
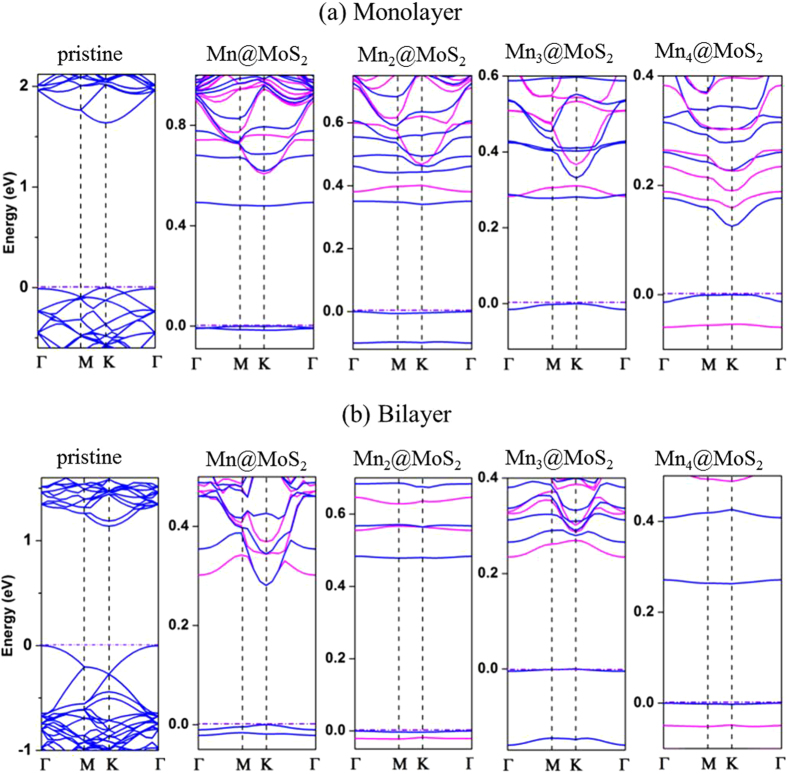
Spin-polarized band structures of the Mn_n_ adsorbed MoS_2_ systems. (**a**) Band structures of the Mn_n_@MoS_2_ pizzas. (**b**) Band structures of the Mn_n_@MoS_2_ sandwiches. The blue and magenta lines represent the spin-up and spin-down components, respectively. The horizontal dash-dot lines indicate the Fermi level. The band structures of the pristine hosts are depicted at the very left column for comparison purpose.

**Figure 4:**
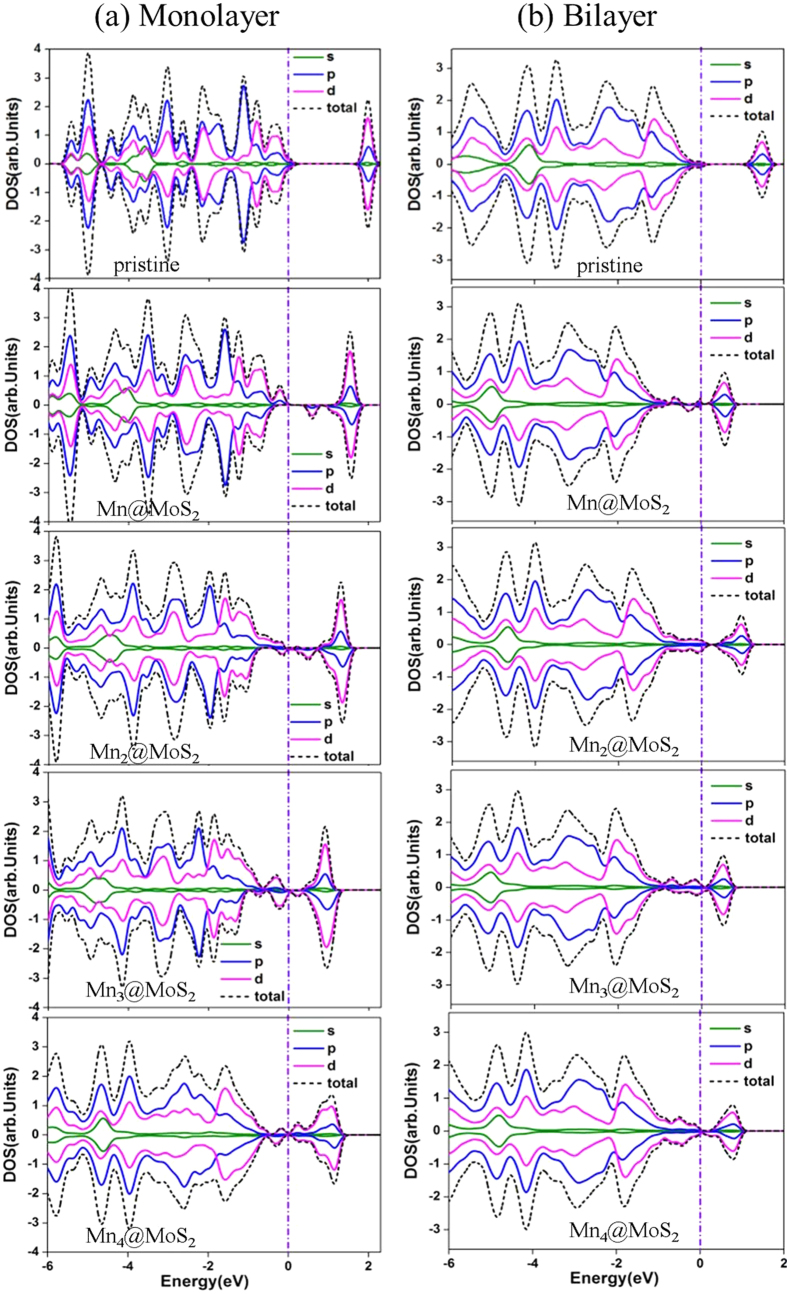
Electronic partial density of states (PDOS) of the Mn_n_ adsorbed MoS_2_ systems. (**a**) The PDOS of spin-up (positive) and spin-down (negative) electrons of the Mn_n_@MoS_2_(M) pizzas. (**b**) The PDOS of spin-up (positive) and spin-down (negative) electrons of the Mn_n_@MoS_2_(B) sandwiches. The PDOS is obtained by Gaussian extension applied to the eigenvalues with a broadening width of 0.1 eV. The vertical dash-dot lines indicate the Fermi level.

The band structures of the Mn_n_@MoS_2_(M&B) complexes were plotted in Fig. 3 for the lowest-energy structures. These from the pristine monolayer and bilayer MoS_2_ were also given for comparison purposes. In the monolayer, a direct bandgap was found to have energies of 1.69 eV and 1.89 eV in our GGA and Heyd-Scuseria-Ernzerhof (HSE06) calculations implemented in CASTEP package^45^,^46^,^47^. The values are in good agreement with previous studies^9^,^48^,^49^,^50^,^51^,^52^,^53^,^54^,^55^. Although GGA at the PBE level calculations typically underestimates this bandgap, there is no difference between the GGA and HSE06 evaluations of the bandgap types. As Fig. 3 shows, the embedment of the Mn_n_ clusters inserts additional defect states within the pristine MoS_2_ bandgap. The valence band maximum (VBM) and conduction band minimum (CBM) are primary from the 3*d* orbitals of the Mn_n_ clusters. The partial density of states (PDOS) of Mn_n_@MoS_2_(M&B) in Fig. 4 clarifies theses defect states are from the Mn_n_ clusters near the VBM and the CBM. Compared with the pristine MoS_2_ cases, Fermi energy shifts from the VBM towards the CBM with the increase of the Mn numbers. Figure 4 also shows that shapes of the total density of states for α electron (spin-up) and β electron (spin-down) near the Fermi energy are quite different in the contributions of the magnetism of the Mn_n_@MoS_2_. The bandgap of the pristine MoS_2_ is evidently reduced due to the absorptions of small TM clusters. Such a reduction can significantly affect material optical and transport properties. From the values listed in Table 3, the bandgap of the ‘pizzas’ decreases gradually with the successive Mn atoms. However, odd-even oscillation emerges again in bilayer system similar to its magnetic properties.

**Figure 5:**
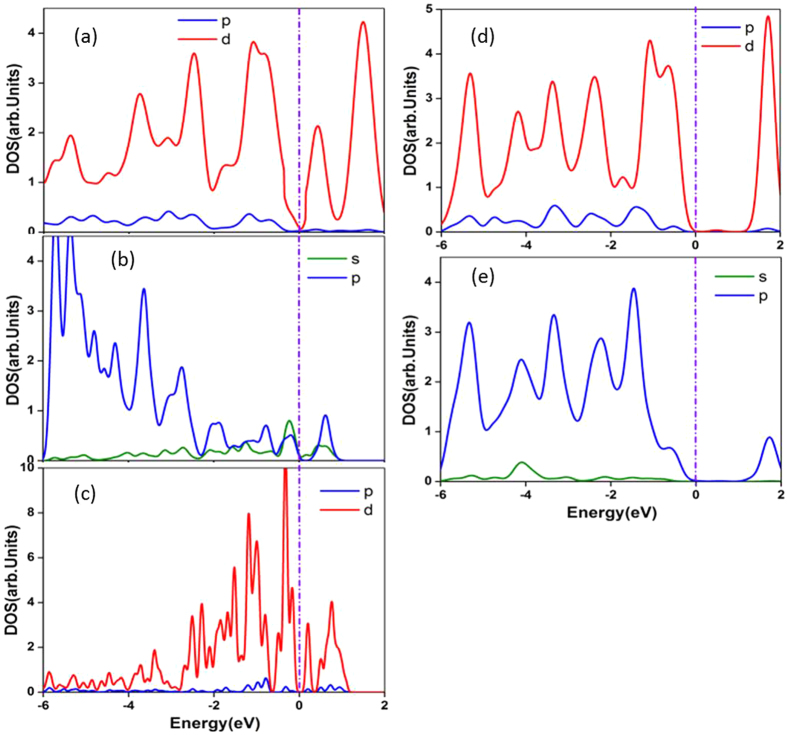
The PDOS of atoms in the Mn_3_@MoS_2_(M) complex. (**a**) Participator Mo atom near the Mn cluster. (**b**) Participator S atom contracting with Mn cluster. (**c**) Mn atom. (**d**) Spectator Mo atom away from the Mn cluster. (**e**) Spectator S atom away from Mn cluster. The illustration is the same as that in Fig. 4.

As the Mn clusters are adsorbed on MoS_2_, there is obvious hybridization between the atomic orbitals of the guest atom Mn and host atom S. We take the PDOS plots of Mo, S, and Mn atoms of the Mn_3_@MoS_2_(M) as an example (see Fig. 5) to explicate the hybridization. Several sharp peak superpositions originate from the PDOS for *d* orbital of Mn and *p* orbital of S in the S-Mn bond below the Fermi level. And the PDOS for Mo and S atoms in S-Mo bond close to the Mn cluster is quite different from these spectator Mo and S atoms far away the Mn cluster. Similar behavior is observed in all other Mn_n_@MoS_2_ systems.

Types of bands can be switched through the present doping route. A transition from an indirect to a direct bandgap in pristine MoS_2_ are found when the thickness is reduced from bilayer to a monolayer, in agreement with previous experimental reports^9^,^56^ and theoretical results^57^,^58^. After the Mn_n_ clusters were introduced to the host MoS_2_ ‘crusts’, the Mn_n_@MoS_2_(M) pizza (n = 1,4) bandgaps keep direct as their host’s. However, the bandgap turns to indirect when n = 2,3 as shown in Fig. 3a. Excitingly, in the case of the Mn_1_@MoS_2_(B) ‘sandwich’, the indirect bandgap of the bilayer host was switched to a direct bandgap. The CBM and VBM are both aligned at the ĸ point. Such a direct band structure is similar to the monolayer’s ones, which have been considered as the crucial origin of the ILC unique material properties. The result indicates the bandgap of pristine MoS_2_ can be operated from an indirect to direct or direct to indirect bandgap by adsorbing small TM clusters like Mn_n_. This provides new opportunities for controlling electronic structures in nanoscale materials with novel optical behaviors.

Ranging from 0.053 to −0.215 and −0.32 to −1.715 au in the ‘pizza’ and ‘sandwiches’ systems, the net charge on the impurity Mn clusters clearly shows charge transfers between the ‘toppings’ and ‘fillings’, and the S atoms in the ‘crusts’ and ‘slices’. This leads high stabilities of the ‘pizza’ and ‘sandwiches’ following the partially ionic-like bonding of the Mn−S interaction through the charge transfers. Except for the Mn_1_@MoS_2_(M), charge transfers occur from the S atoms to the Mn atoms resulting in negative charges of the Mn_n_ clusters. For the Mn_n_@MoS_2_(B), increases of the net charge values on the Mn clusters were found, illustrating enhancements of the sandwiches structures as the successive add-on dopant. The charge transfers between the Mn clusters and host MoS_2_ are one reason of the reducing magnetic moment of Mn_n_@MoS_2_ from the isolate Mn clusters, while strong hybridizations of the sulfur atoms in the MoS_2_ with the *d* states of the Mn cluster atoms is counted as another.

### Thermostabilities

**Figure 6:**
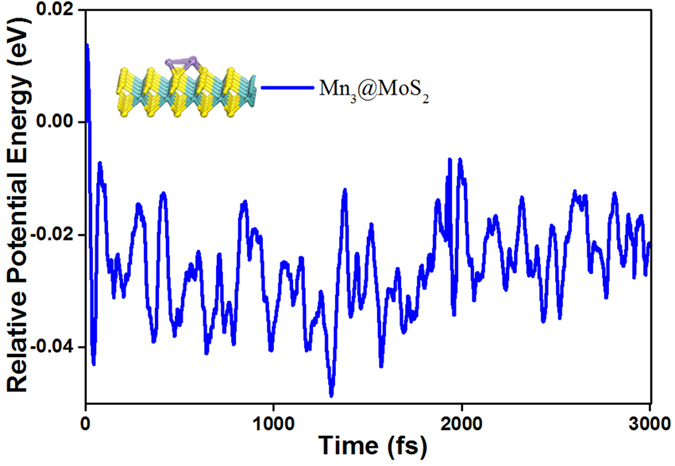
Relative potential energy (eV) of the lowest-energy structures Mn_3_@MoS_2_(M). The simulation time was set to 3 ps at a step interval of 1 fs in the molecular dynamics simulation.

The thermodynamic stability was tested by using the Born−Oppenheimer molecular dynamics simulation implemented in the DMOL3 code at room temperature (T = 300 K). A sample of the dynamic simulations is shown in Fig. 6 for the Mn_3_@MoS_2_(M) pizza case. It is clear that the relative potential energy remains unchanged within the selected time scale. The ground-state structure is stable at room temperature. Such a thermostability is in line with the experimental evidences of the Au adsorbed MoS_2_ monolayer^59^ and the latest results of the water intercalated organic counterpart of the graphene^35^.

In conclusion, we have presented a new strategy of tailoring the inorganic layered crystal to the magnetic semiconductors by introducing magnetic clusters as adsorbates. Geometric optimizations show that the small clusters prefer to follow the host alignments to enhance the complex stabilities. The magnetic and electronic structures were thoroughly explored. It is found that the system magnetic properties and electronic structures can be manipulated by careful selections of the ‘pizza’ and ‘sandwich’ recipes. Moreover, switches between the direct and indirect bandgaps of the adsorbed MoS_2_ complexes were revealed. Benefiting from the uniqueness of the clusters and inorganic layered crystals, it is hoped that the present work will be served as a prototype in combinations of the cluster and layered crystal sciences, and boost their applications in the semiconducting scopes.

## Methods

All calculations were performed by using the DMOL3 package^60^,^61^. Results from the present package were cross checked with the calculations from CASTEP package. A relativistic semi-core pseudopotential was employed for the spin-unrestricted calculations with double-numerical basis where *d* polarization functions (DNP) were included. Generalized gradient approximation in the Perdue−Burke−Ernzerhof (PBE) functional form was chosen^62^. The effect of van der Waals interactions was introduced explicitly through an empirical correction scheme proposed by Ortmann, Bechstedt, and Schmidt^63^. The quality of the self-consistent field (SCF) convergence tolerance was set as “fine”. A convergence criterion of 1 × 10^−5^ hartree was applied on the total energy and electron density, 2 × 10^−3^ hartree/Å on the gradient, and 5 × 10^−3^ Å on lattice displacements. The 5 × 5 supercells were constructed from 75 atoms of 25 Mo atoms and 50 S atoms for the monolayer, and 150 atoms including 50 Mo atoms and 100 S atoms for the bilayer. A vacuum region of 25 Å was selected in the z-direction to exclude mirror interactions between neighboring images. The Brillouin Zone integrations were carried out on a 10 × 10 × 1 Monkhorst−Pack k-points grad for the geometry optimizations, and a 15 × 15 × 1 k-points grad for the band and density of states (DOS) properties. To elucidate system magnetic properties, we carried out a detailed calculation for each possible spin multiplicity (SM) ranging from 1 to 21 of the Mn_n_ (n = 1–4) adsorbed MoS_2_ complexes.

## Additional Information

**How to cite this article**: Zhang, M. *et al*. Magnetic MoS_2_ pizzas and sandwiches with Mn_n_ (n=1–4) cluster toppings and fillings: A first-principles investigation. *Sci. Rep.* **6**, 19504; doi: 10.1038/srep19504 (2016).

## Supplementary Material

Supplementary Information

## Figures and Tables

**Table 1:** Adsorption energies *E*_ad_ (in eV) and bond lengths *d* (in Å) of the Mn_n_ (n = 1–4) clusters adsorbed on the monolayer and bilayer MoS_2_.

Layer Number	System Name	Optimized bond distance (Å)			*E*_ad_ (eV)
		*d*_Mn-Mn_	nearest *d*_Mn-S_	farthest *d*_Mo-S_	
0	Mn_2_ cluster	2.633	—	—	—
	Mn_3_ cluster	2.791	—	—	—
	Mn_4_ cluster	2.764	—	—	—
1	pristine	—	—	2.437	—
	Mn_1_@MoS_2_	—	2.207	2.610	−1.631
	Mn_2_@MoS_2_	2.877	2.116	2.654	−2.897
	Mn_3_@MoS_2_	2.720	2.181	2.556	−4.207
	Mn_4_@MoS_2_	2.631	2.206	2.531	−4.725
2	pristine	—	—	2.432	—
	Mn@MoS_2_	—	2.327	2.472	−2.791
	Mn_2_@MoS_2_	2.917	2.086	2.578	−5.013
	Mn_3_@MoS_2_	2.956	2.238	2.457	−6.527
	Mn_4_@MoS_2_	2.940	2.087	2.469	−8.787

The MoS_2_ layer number was set 0 for the free manganese clusters.

**Table 2:** **Magnetic moments** ***M*** (***μ***_**B**_**) of the Mn**_**n**_ **cluster adsorbed MoS**_**2**_ **complexes.**

Monolayer	*M*(*μ*_B_)	Δ*E*(eV)	Bilayer	*M*(*μ*_B_)	Δ*E*(eV)
Mn@MoS_2_	1	0.62	Mn@MoS_2_	1	0.25
	3	0		3	0
	5	0.23		5	0.15
Mn_2_@MoS_2_	0	—	Mn_2_@MoS_2_	0	0.06
	2	0.09		2	0
	4	0.31		4	0.16
	6	0		6	0.15
	8	0.07		8	0.63
	10	0.32		10	1.29
Mn_3_@MoS_2_	1	0.21	Mn_3_@MoS_2_	1	0.09
	3	0.31		3	0
	5	0.93		5	0.13
	7	0.42		7	0.18
	9	0		9	0.1
	11	0.02		11	0.21
	13	0.49		13	0.36
	15	0.89		15	0.55
Mn_4_@MoS_2_	0	0.24	Mn_4_@MoS_2_	0	0.05
	2	0.07		2	0
	4	0		4	0.26
	6	0.28		6	0.36
	8	0.31		8	0.55
	10	0.16		10	—
	12	0.04		12	0.88
	14	0.23		14	1.39
	16	0.66		16	1.95
	18	1.03		18	2.59
	20	1.38		20	—

Metastable isomers of other magnetic moments have different relative energies of ∆*E* (in eV) with respect to the most stable ones. The hyphen (—) means the structure is not converged during the optimization.

**Table 3:** Charge transfer and local magnetic moments of the Mn_n_ cluster adsorbed MoS_2_ complexes.

System	magnetic moment (*μ*_B_)			total	charge	bandgap
	Mn	Mo	S		Mn	
pristine	—	0	0	0	—	1.687
Mn@MoS_2_(M)	3.26	−0.132	−0.128	3	0.053	0.463
Mn_2_@MoS_2_(M)	6.706	−0.502	−0.204	6	−0.07	0.327
Mn_3_@MoS_2_(M)	9.578	−0.362	−0.216	9	−0.175	0.245
Mn_4_@MoS_2_(M)	4.579	−0.51	−0.069	4	−0.215	0.137
pristine	—	0	0	0	—	1.144
Mn@MoS_2_(B)	2.881	0.027	0.092	3	−0.32	0.272
Mn_2_@MoS_2_(B)	2.042	−0.016	−0.026	2	−0.771	0.490
Mn_3_@MoS_2_(B)	3.195	−0.224	0.029	3	−0.808	0.245
Mn_4_@MoS_2_(B)	1.953	0.147	−0.1	2	−1.715	0.276

Mulliken Charge (in au) were counted on the Mn atoms, local magnetic moment (in *μ*_B_) on the guest Mn clusters, host Mo and S atoms. Total magnetic moment (in *μ*_B_) and bandgap (in eV) of the Mn_n_ clusters absorbed on monolayer and bilayer MoS_2_ per supercell were also tabulated herein.
