# Supplementary material for: Magnetic MoS2 pizzas and sandwiches with Mnn (n = 1–4) cluster toppings and fillings: A first-principles investigation
Source: Sci Rep. 2016 Jan 18;6:19504. doi: 10.1038/srep19504 (PMC4725988; doi:10.1038/srep19504)
Supplement: Supplementary Information [file srep19504-s1.pdf]

Supplementary Information

For

Magnetic MoS_2 pizzas and sandwiches with Mn_n (n=1-4) cluster toppings and fillings: A first-principles investigation

Meng Zhang[1], Zhongjia Huang[2], Xiao Wang[1], Hongyu Zhang[1], Taohai Li[3], Zhaolong Wu[1] ,Youhua Luo[1*], and Wei Cao[4*]

[1] Department of Physics, East China University of Science and Technology, Shanghai 200237, China

[2] School of Mechanical and Automotive Engineering, Anhui Polytechnic University, Wuhu 241000, China

3 College of Chemistry, Key Lab of Environment Friendly Chemistry and Application in Ministry of Education, Xiangtan University, Xiangtan, 411105, China

[4] Research Centre for Molecular Materials, University of Oulu, P.O. Box 3000, FIN-90014, Finland

* To whom correspondence should be addressed. E-mail: yhluo@ecust.edu.cn (Y.L.); wei.cao@oulu.fi (W.C.)

Table of Contents

1. Figure S1. Optimized geometries of the small Mn_n (n=1-4) isomers.
2. Table S1. Atomic magnetic moments (in μ_B) of the Mn_n cluster adsorbed MoS_2 complexes.

Figure S1. Lowest-energy structures and low-lying isomers with magnetic moments and relative energies (in eV) of small Mn_n (n=1-4) clusters. The symmetry type is given at 0.01 Å tolerances.

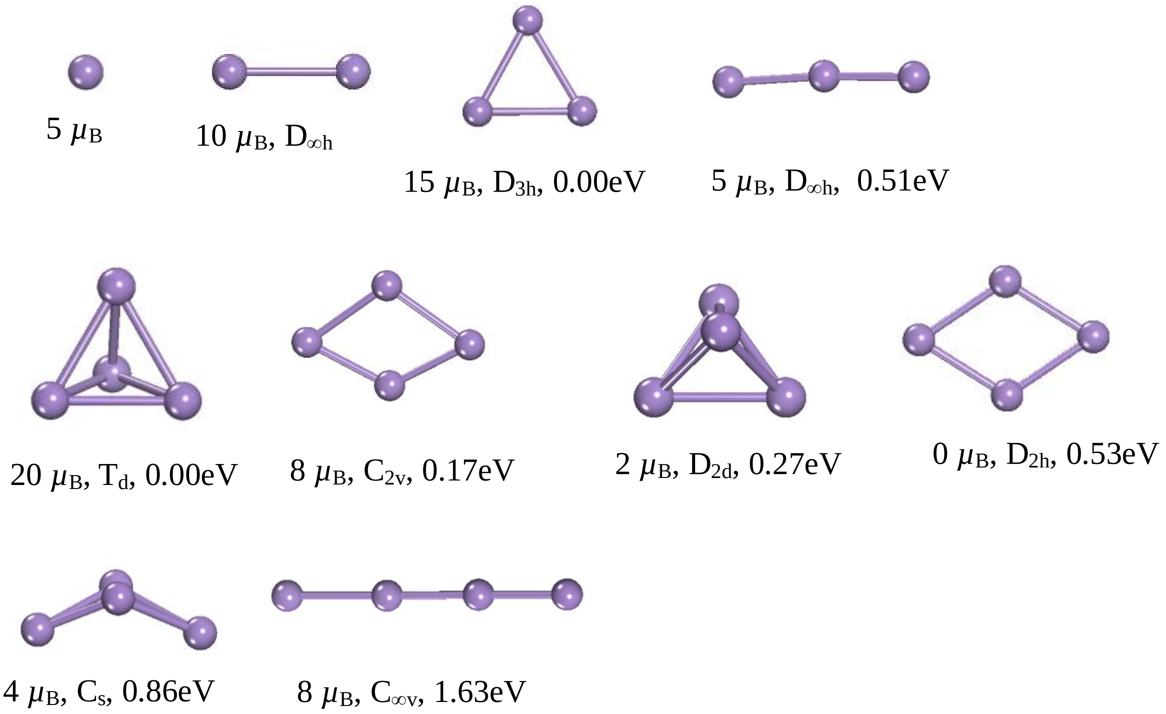

Table S1. Atomic magnetic moments (in μ_B) on each Mn atom of the Mn_n cluster adsorbed MoS_2 complexes. The Mn atoms are labeled in the structures.

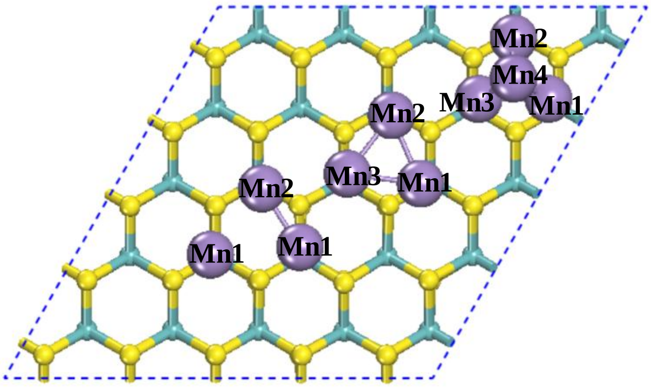

Monolayers

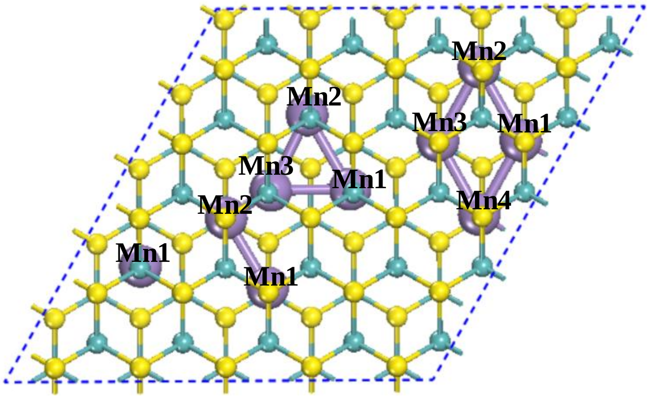

Bilayers

System	magnetic moment (μ_B)		
	Mn atoms		Mn clusters
$Mn@MoS_2$(M)	Mn1	3.260	3.260
$Mn_2@MoS_2$(M)	Mn1	3.403	6.706
	Mn2	3.303	
$Mn_3@MoS_2$(M)	Mn1	3.188	9.578
	Mn2	3.200	
	Mn3	3.190	
$Mn_4@MoS_2$(M)	Mn1	2.944	4.579
	Mn2	2.944	
	Mn3	2.901	
	Mn4	-4.210	
$Mn@MoS_2$(B)	Mn1	2.881	2.881
$Mn_2@MoS_2$(B)	Mn1	2.337	2.042
	Mn2	-0.295	
$Mn_3@MoS_2$(B)	Mn1	2.945	3.195
	Mn2	-2.698	
	Mn3	2.948	
$Mn_4@MoS_2$(B)	Mn1	0.363	1.953
	Mn2	-0.178	
	Mn3	0.348	
	Mn4	1.420	
